# Supplementary material for: Endocrine lineage biases arise in temporally distinct endocrine progenitors during pancreatic morphogenesis
Source: Nat Commun. 2018 Aug 22;9:3356. doi: 10.1038/s41467-018-05740-1 (PMC6105717; doi:10.1038/s41467-018-05740-1)
Supplement: Supplementary file 1 — Supplementary Information [file 41467_2018_5740_MOESM1_ESM.pdf]

Supplementary Information

Endocrine lineage biases arise in temporally distinct endocrine progenitors during pancreatic morphogenesis

Marissa A. Scavuzzo*, Matthew C. Hill*, Jolanta Chmielowiec, Diane Yang, Jessica Teaw, Kuanwei Sheng, Yuelin Kong, Maria Bettini, Chenghang Zong, James F. Martin, and Malgorzata Borowiak

Contents

1. Supplementary Figures 1-18
2. Supplementary Tables 1-2

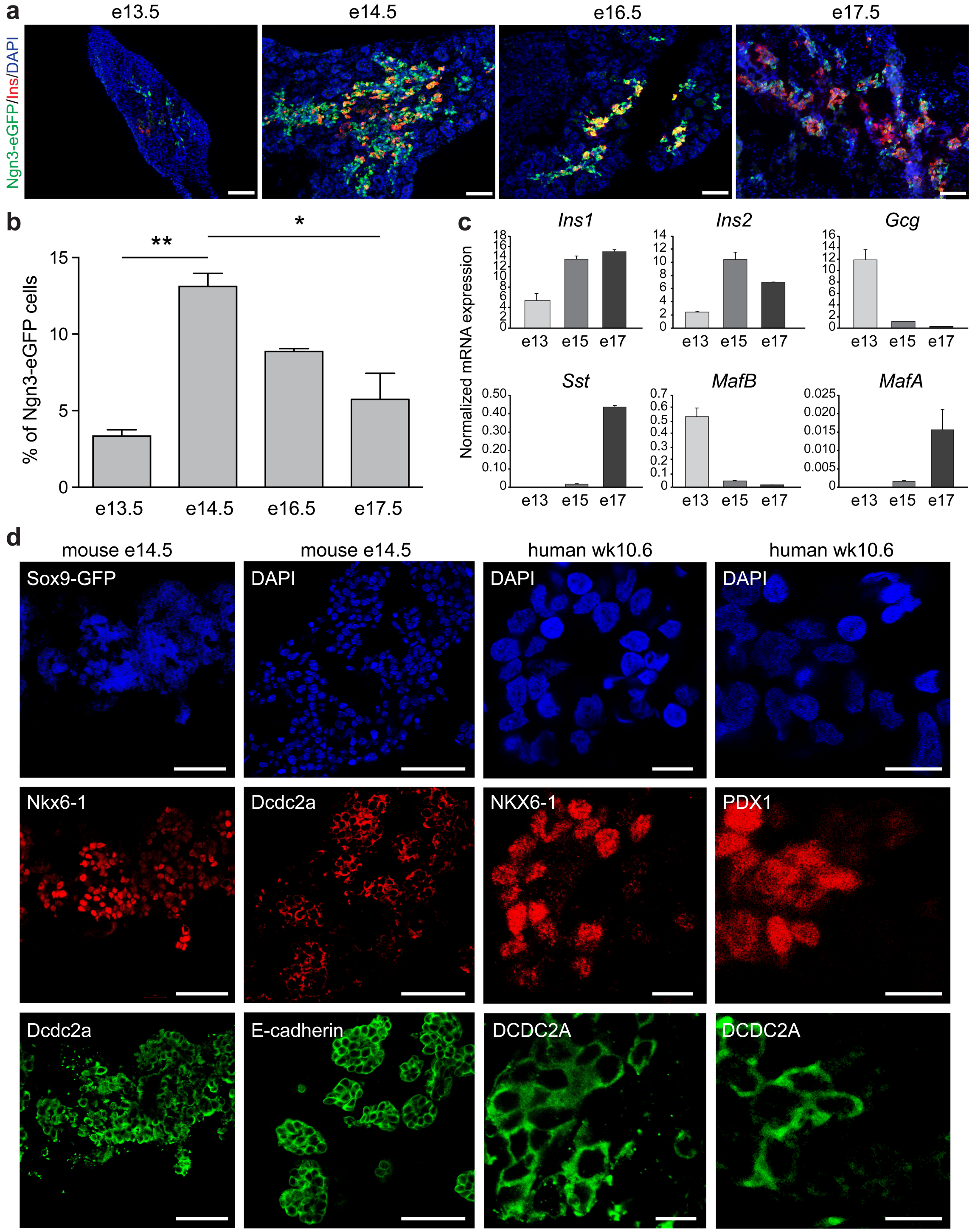

Supplementary Figure 1. Ngn3+ cell formation peaks between e14.5 and e16.5.

a. Endocrine progenitors are abundant at e14.5 and e16.5. Immunostaining of Ngn3-eGFP (green), Ins (red), and DAPI (blue) at e13.5, e14.5, e16.5, and e17.5. Representative images from 2 biological replicates are shown.

b. Quantification of Ngn3-eGFP+ cells relative to DAPI. N=2 mice per timepoint. Error bars are SEM. *p<0.05, **p<0.01 by one-way ANOVA with multiple comparisons.

c. Gene expression changes from e13.5 to e17.5 in the mouse pancreas. qPCR of endocrine markers. Error bars are SEM. N=3 mice per timepoint, N=2 technical replicates. Samples are normalized to *Gapdh*.

d. Single channel images from Fig. 1g and 1h. Scale bars for mouse = 50um. Scale bars for human = 10um.

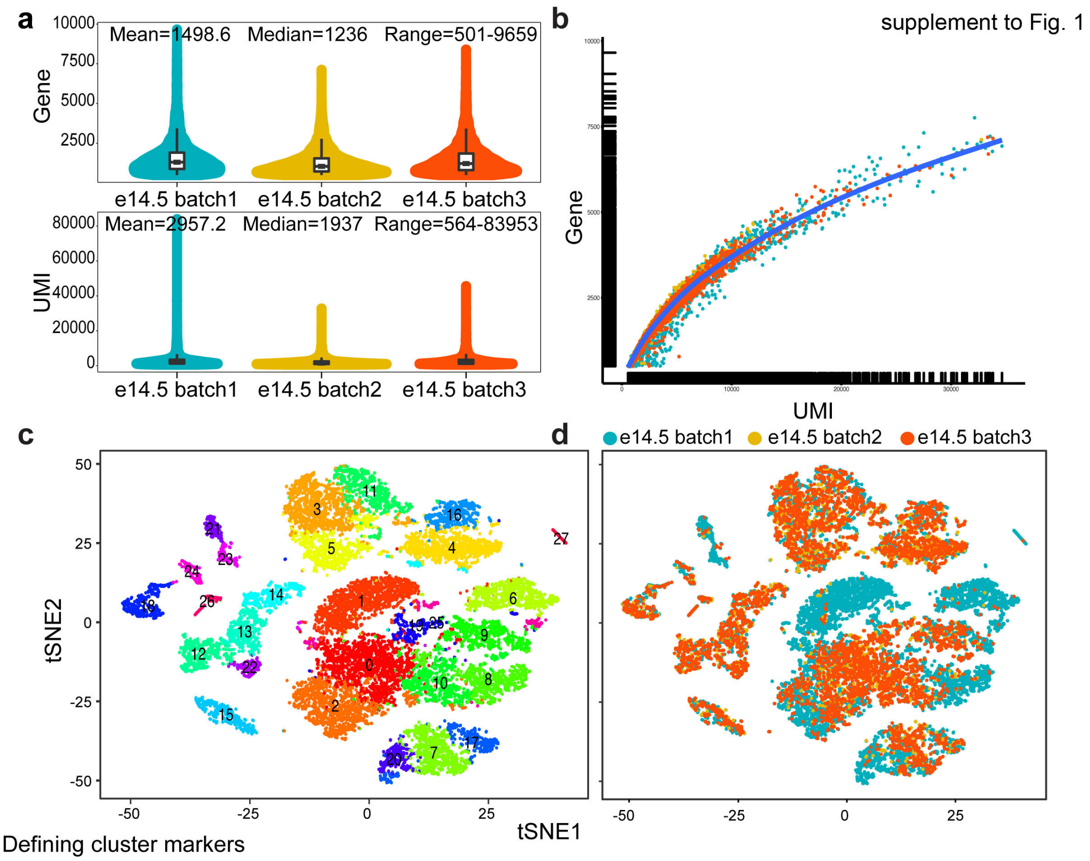

e Defining cluster markers

Alpha	Beta	BP	Endo	EP	Eryth	Hepato	MΦ	Mast	Mes	MesO	Neuron	PaSC	TC
Gcg	*Ins2*	*Spp1*	*Pecam1*	*Ngn3*	*Hbb-bt*	*Afp*	*Mrc1*	*Cpa3*	*Itm2a*	*Upk3b*	*Phox2b*	*Nes*	*Rbpjl*
Arx	*Ins1*	*Sox9*			*Hemgn*	*Alb*	*Lyz2*	*Cd52*	*Col3a1*	*Lrrn4*	*Ascl1*	*Vim*	*Ptf1a*
Pou3f4	*Nkx6.1*	*Nkx6.1*						*Nkg7*	*Vim*	*Aldh1a2*	*Hand2*	*Vegfa*	*Nr5a2*
									Nkx2.5		*Gap43*	*Ncam1*	*Cpa1*
									Col1a2		*Phox2a*	*Pdgfra*	
									Ptn		*Tlx2*	*Pdgfrb*	
											Tbx3		

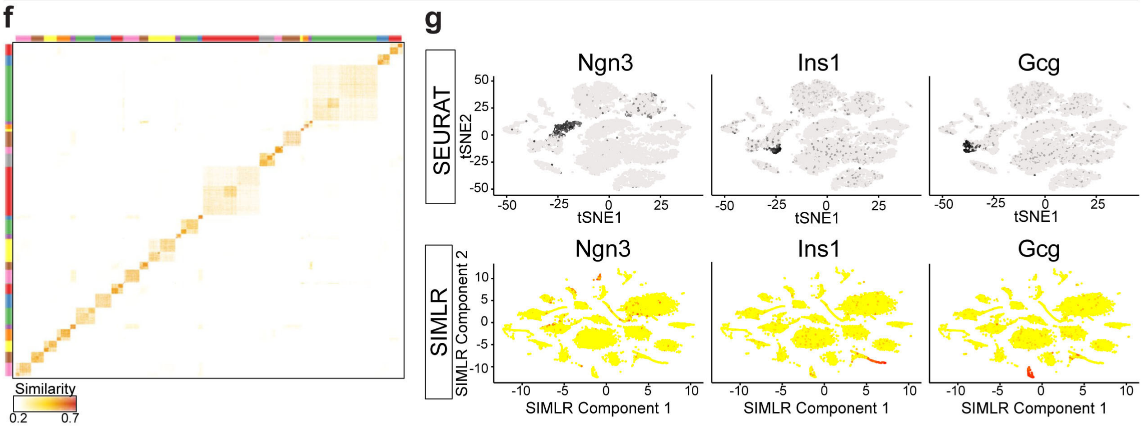

Supplementary Figure 2. Droplet-based RNA-sequencing of the e14.5 pancreas.

a. Average number of genes and UMIs for the three Drop-seq runs consisting of 39 e14.5 pancreata from 3 litters and 15,228 single cells.

b. Strong correlation between genes and UMIs.

c. Cluster formation prior to merging non-distinct groups. tSNE representation of single cell data showing original clusters before merging non-distinct clusters (from likelihood-ratio test for single cell gene expression). Unique clusters possessed at least 5 markers with an average expression greater than 0.8.

d. Tracing of individual scRNA-seq batches. Batch 1 was composed of 14 embryos from a single litter, batch 2 from 13 embryos from a single litter, and batch 3 from 12 embryos from a single litter. Clusters 1, 6, 8, and 27 are likely from imprecise dissection and inclusion of extra support tissue (1, 6, and 8 are Mes groups; 27 is hepatocytes).

e. Transcripts enriched in clusters used to demarcate their identity.

f. Similarity heatmap of graph-based (Seurat) and kernel-based (SIMLR) clustering algorithms.

g. Seurat maintains endocrine cell developmental relationships compared to SIMLR. Feature plots showing expression of key genes amongst clusters, including EP marker Ngn3, beta cell marker Ins2, and alpha cell marker Gcg.

a

	#	% of total
EPhi	422	3.1%
EPlo	451	3.0%
alpha	465	2.8%
beta	170	1.1%

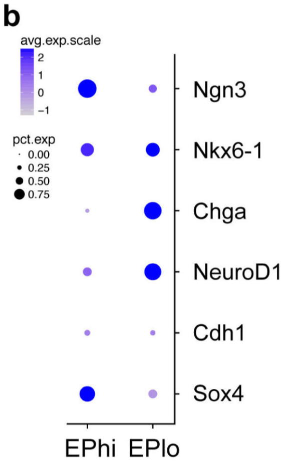

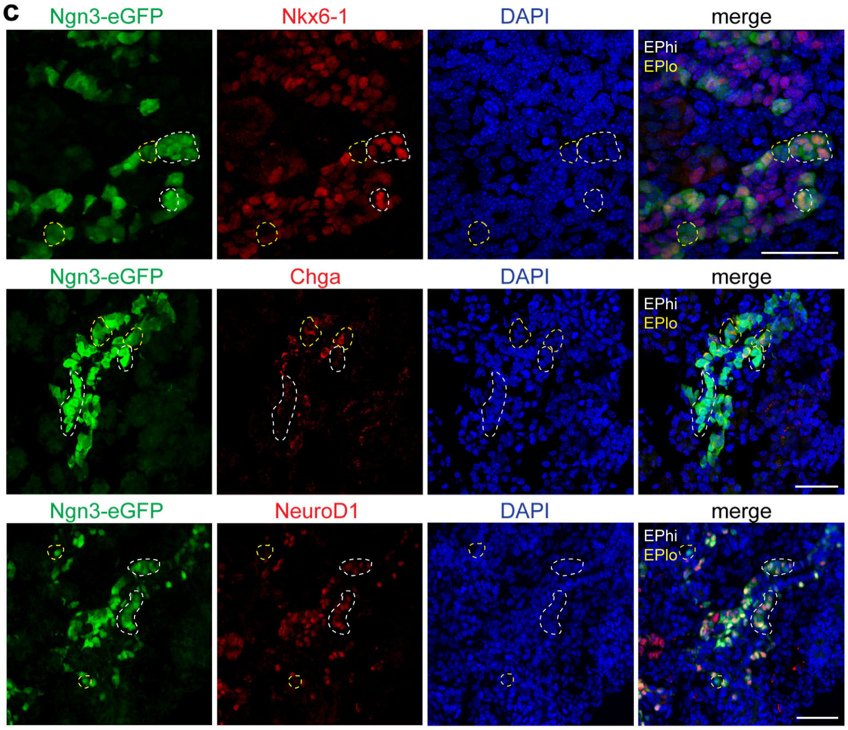

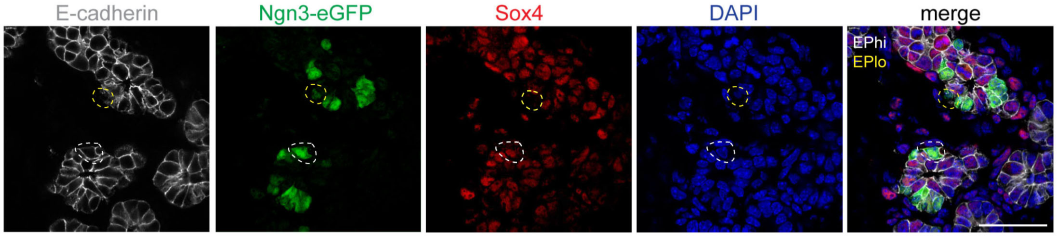

Supplementary Figure 3. Validation of EPhi and EPlo subtypes.

a. Number and percentage of EPhi, EPlo, alpha, and beta cells from Fig. 1b.

b. Dot plot showing select gene expression in EPhi and EPlo clusters.

c. Immunostaining of select markers showing EPhi (circled in white) and EPlo (circled in yellow) cells. Scale bars=50um.

supplement to Fig. 2

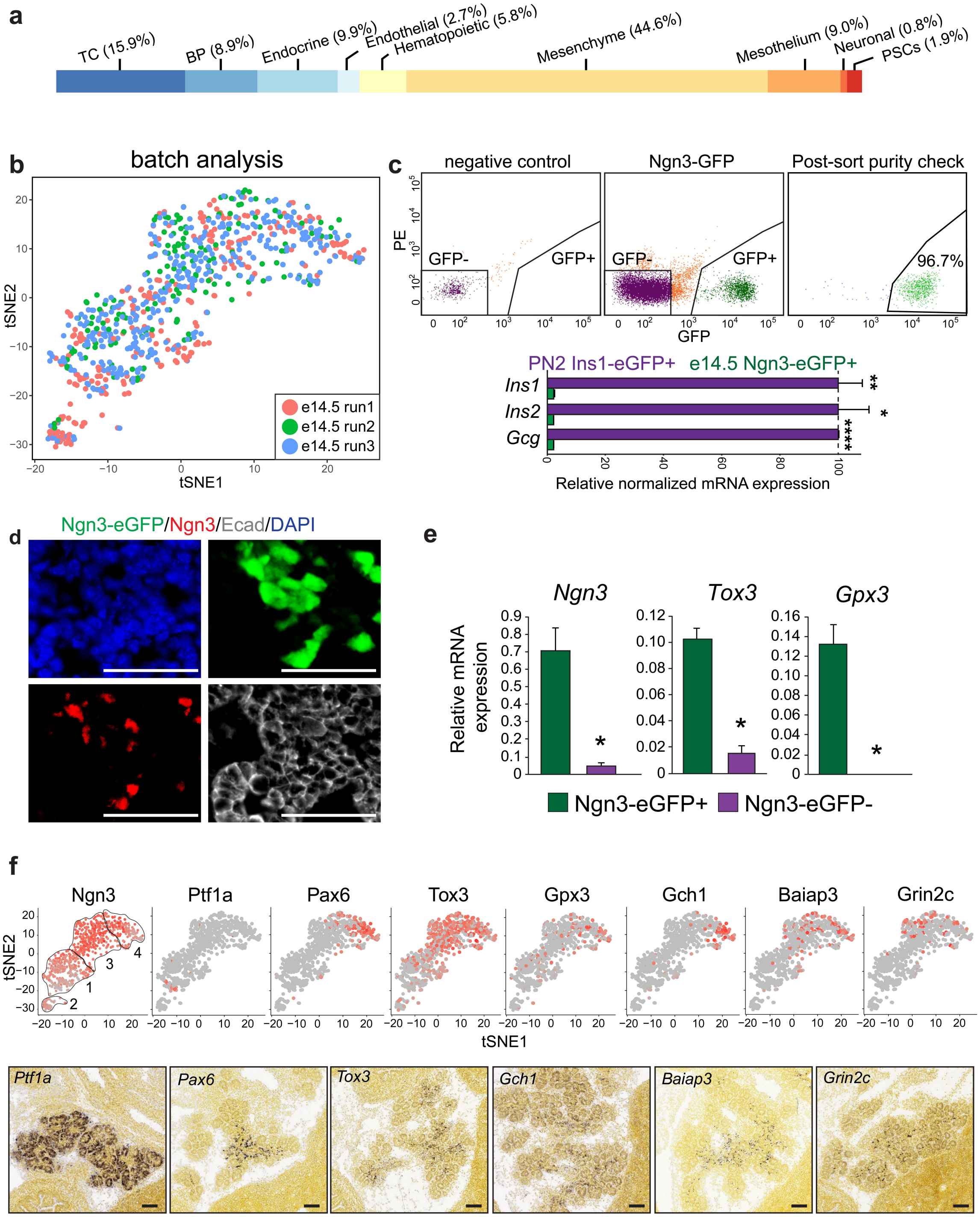

Supplementary Figure 4. Validation of EP subtypes and markers at e14.5.

a. Cellular composition of the e14.5 mouse pancreas as % of total cells. Composition of different endocrine cell types and mesenchymal cell types are highlighted as % of total

cluster. 9.9% of cells are endocrine, with a high percentage (30.8%) of acinar cells at e14.5.

b. tSNE representation of single e14.5 Ngn3+ cell transcriptomes analyzed for batch effects. Cells were derived from three separate litters of mice indicated by color. Each EP subtype is composed of mixed populations represented across all three batches.

c. Fluorescence-activated cell sorting (FACS) plots showing e14.5 Ngn3-eGFP mice. Post-sort purity check of isolated Ngn3-eGFP (GFP) cells, showing cells to be 96.7% eGFP+. qPCR shows the expression of *Ins1, Ins2,* and *Gcg* in e14.5 Ngn3-eGFP+ cells relative to Ins1-eGFP. Samples are normalized to *Gapdh.* N=3 biological replicates, N=2 technical replicates. Error bars are SEM. *$p<0.05$, **$p<0.01$, ****$p<0.001$.

d. Ngn3-eGFP overlaps with Ngn3 protein. Immunostaining of e14.5 Ngn3-eGFP (in green) mouse pancreas co-stained for Ngn3 protein (in red) and epithelium marked by Ecad (in white). Nuclei are marked by DAPI in blue. Scale bar=50um.

e. qPCR showing expression of *Ngn3, Gpx3,* and *Tox3* in e14.5 EPs and non-EPs. Samples are normalized to *Gapdh.* N=3 biological replicates, N=2 technical replicates. Error bars are SEM. *$p<0.05$.

f. Droplet-based RNA-seq revealed pancreatic genes not yet described in embryogenesis. Feature plots show candidate gene expression. RNA *in situ* of candidate EP genes in the e15.5 pancreas discovered by Drop-seq. *Ptf1a* and *Pax6* show exocrine and endocrine cells. Images are from Allen Brain Atlas. Scale bars=198um.

supplement to Fig. 2

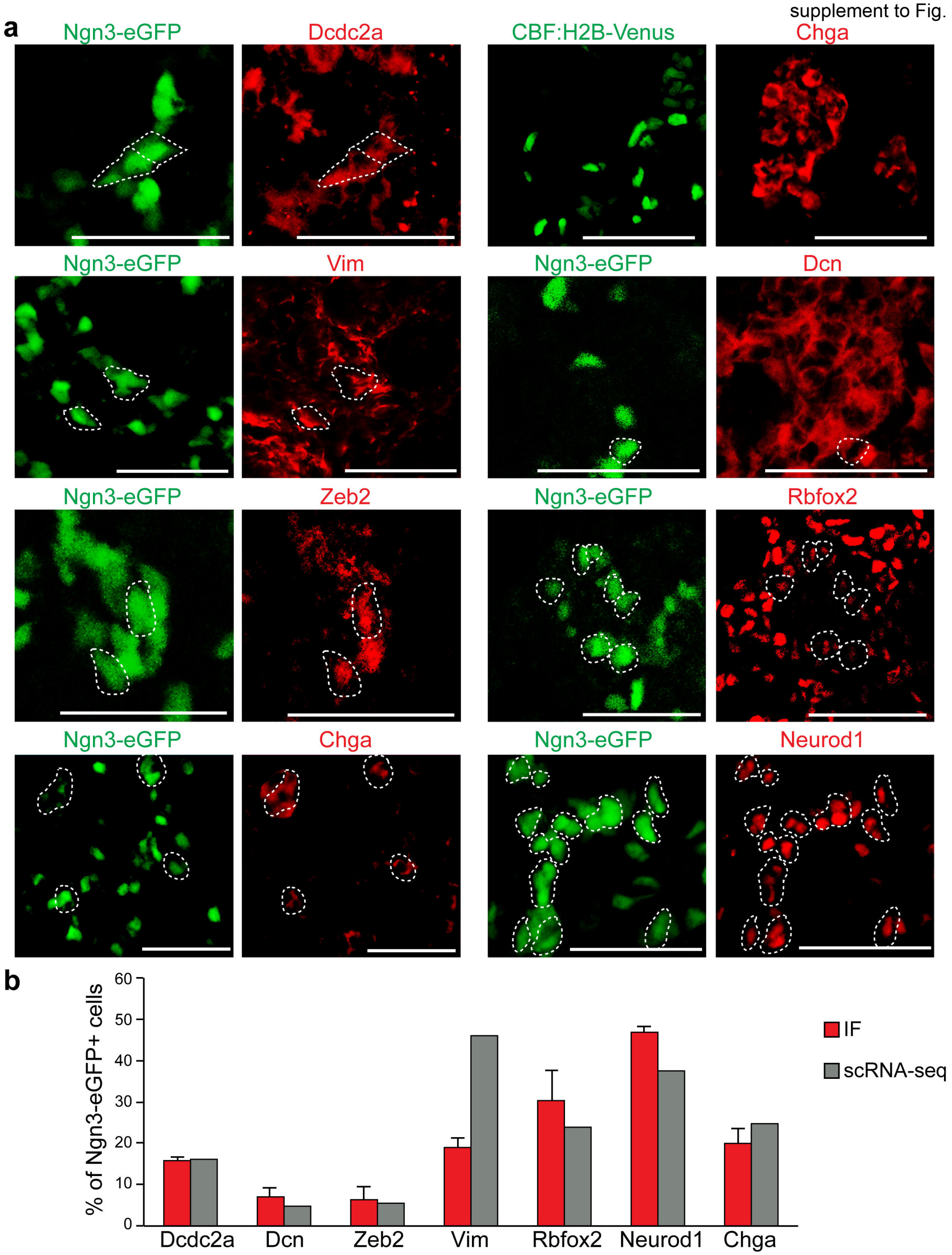

Supplementary Figure 5. ***Ngn3*+ cell subtypes from e14.5.**

a. Single channel images from Fig. 2e. Scale bars = 20um. Representative images from biological replicates are shown (Dcdc2a, Rbfox2, and Neurod1, N=3. Dcn, N=4. Zeb2, Vim, and Chga, N=2).
b. Quantification of Ngn3-eGFP+ cells co-stained with select markers. Dcdc2a, Rbfox2, and Neurod1, N=3. Dcn, N=4. Zeb2, Vim, and Chga, N=2. Gray bars represent scRNA-seq data. Error bars are SEM.

supplement to Fig.2

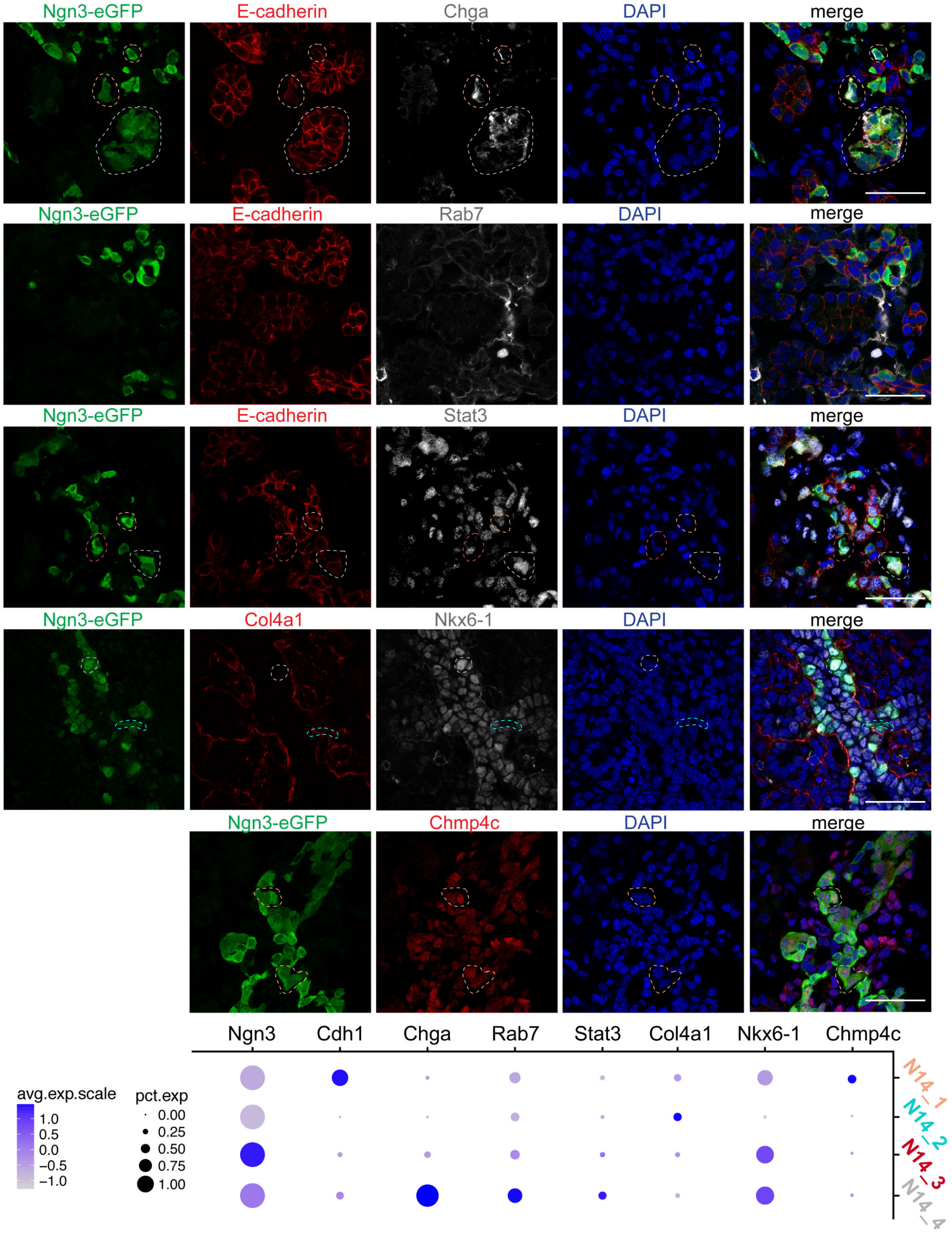

Supplementary Figure 6. Combinatorial immunostaining to validate e14.5 *Ngn3*+ cell subtypes.

Immunostaining of e14.5 mouse pancreatic sections (top). *Ngn3+* subtypes are circled by color (N14_1 in tan, N14_2 in teal, N14_3 in maroon, and N14_4 in gray). Scale bar = 50um. Dot plot showing gene expression in *Ngn3+* cell subtypes (bottom).

supplement to Fig. 2

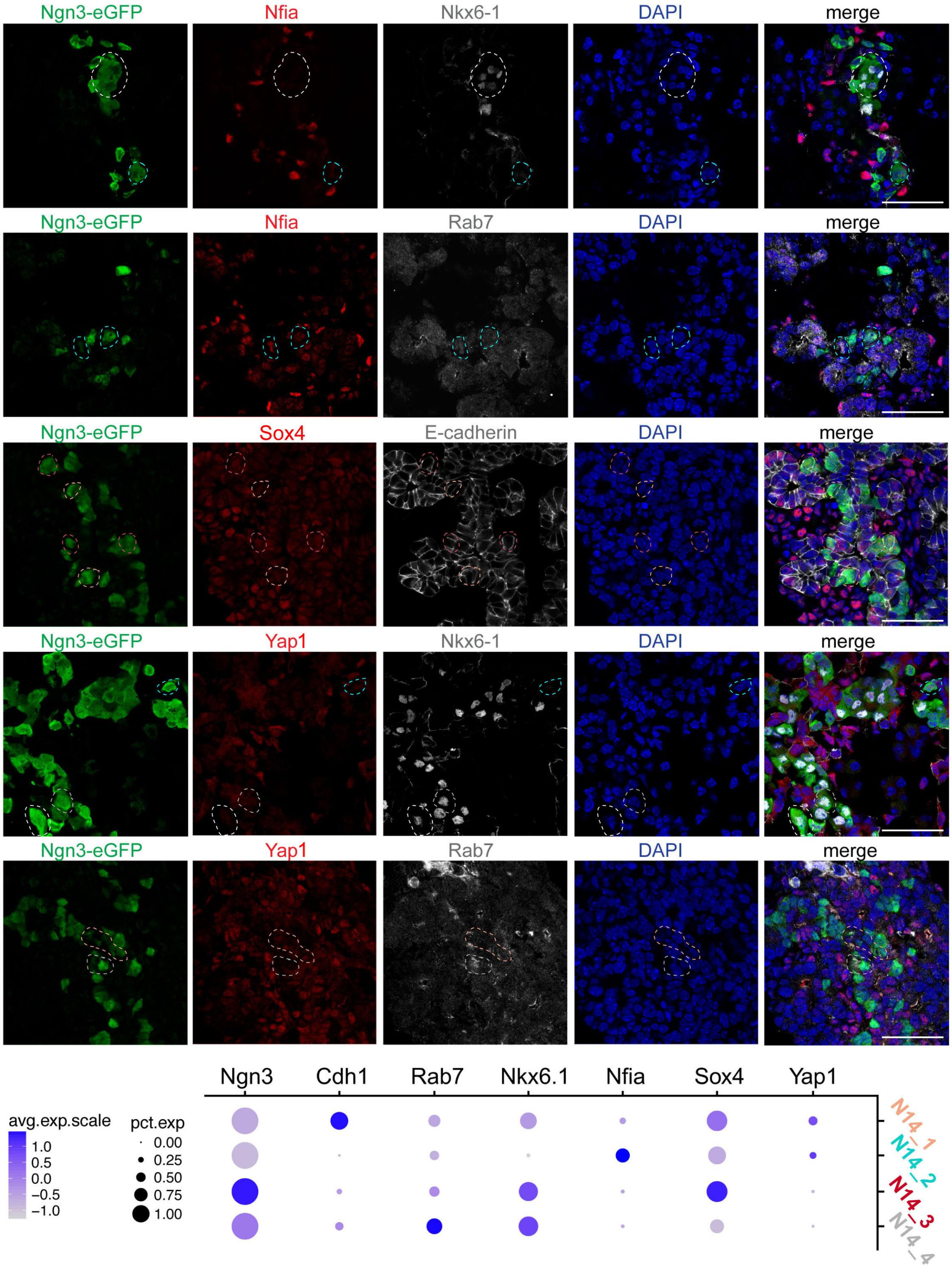

Supplementary Figure 7. Combinatorial immunostaining to validate e14.5 *Ngn3*+ cell subtypes.

Immunostaining of e14.5 mouse pancreatic sections (top). *Ngn3+* subtypes are circled by color (N14_1 in tan, N14_2 in teal, N14_3 in maroon, and N14_4 in gray). Scale bar = 50um. Dot plot showing gene expression in *Ngn3+* cell subtypes (bottom).

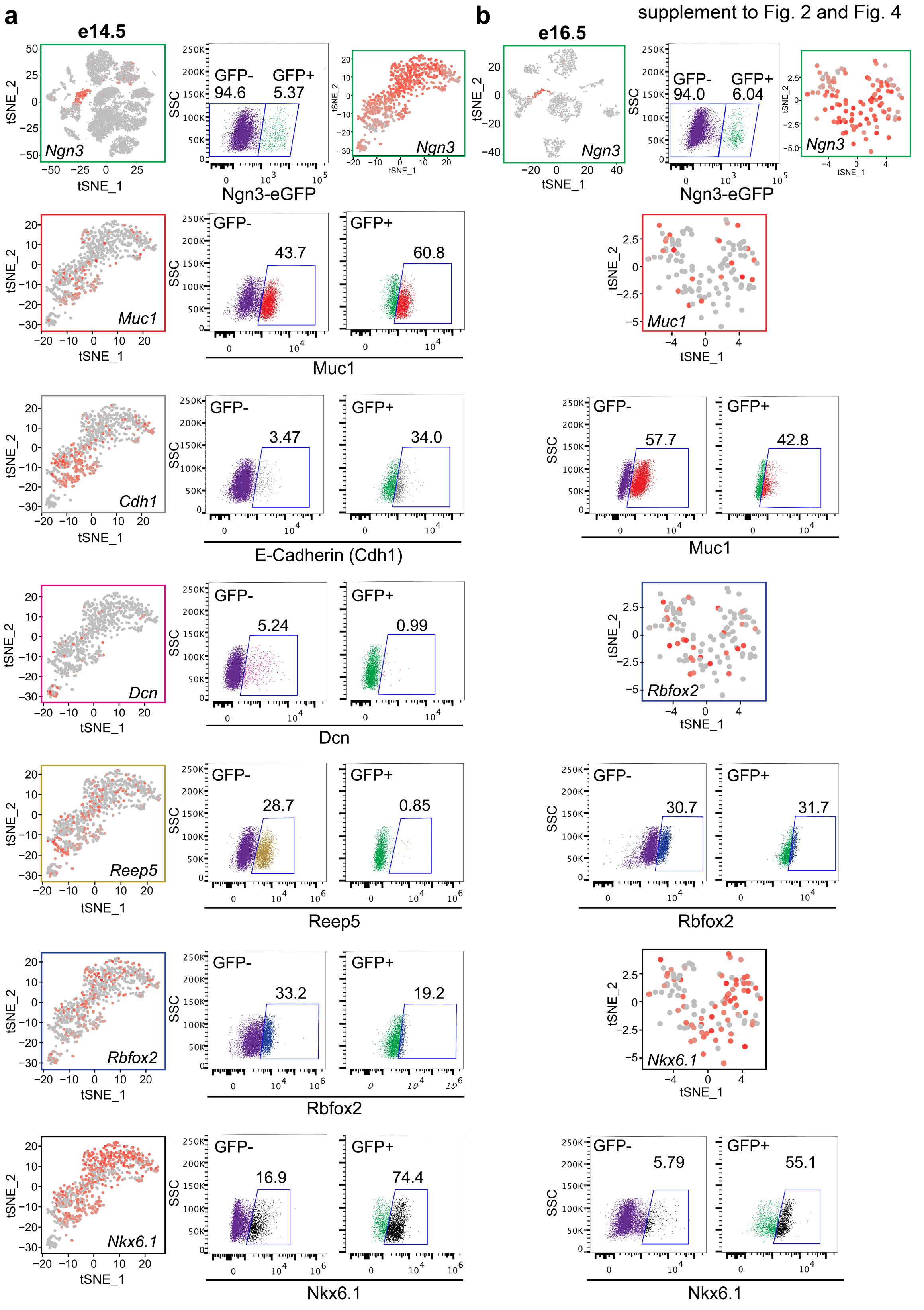

Supplementary Figure 8. Validation of EP subtypes and markers at e14.5 by flow cytometry.

a. Analyses of *Ngn3*+ cell subtype composition by flow cytometry. *Ngn3*+ cells from e14.5 Ngn3-eGFP mice were immunostained for *Ngn3*+ cell subtype genes before gating for Ngn3-eGFP expression and analyzing the percentage of Ngn3-eGFP+ cells co-stained for *Ngn3*+ cell subtype genes.
b. Analyses of *Ngn3*+ cell subtype composition by flow cytometry. EPs from e16.5 Ngn3-eGFP mice were immunostained for *Ngn3*+ cell subtype genes before gating for Ngn3-eGFP expression and analyzing the percentage of Ngn3-eGFP+ cells co-stained for *Ngn3*+ cell subtype genes.

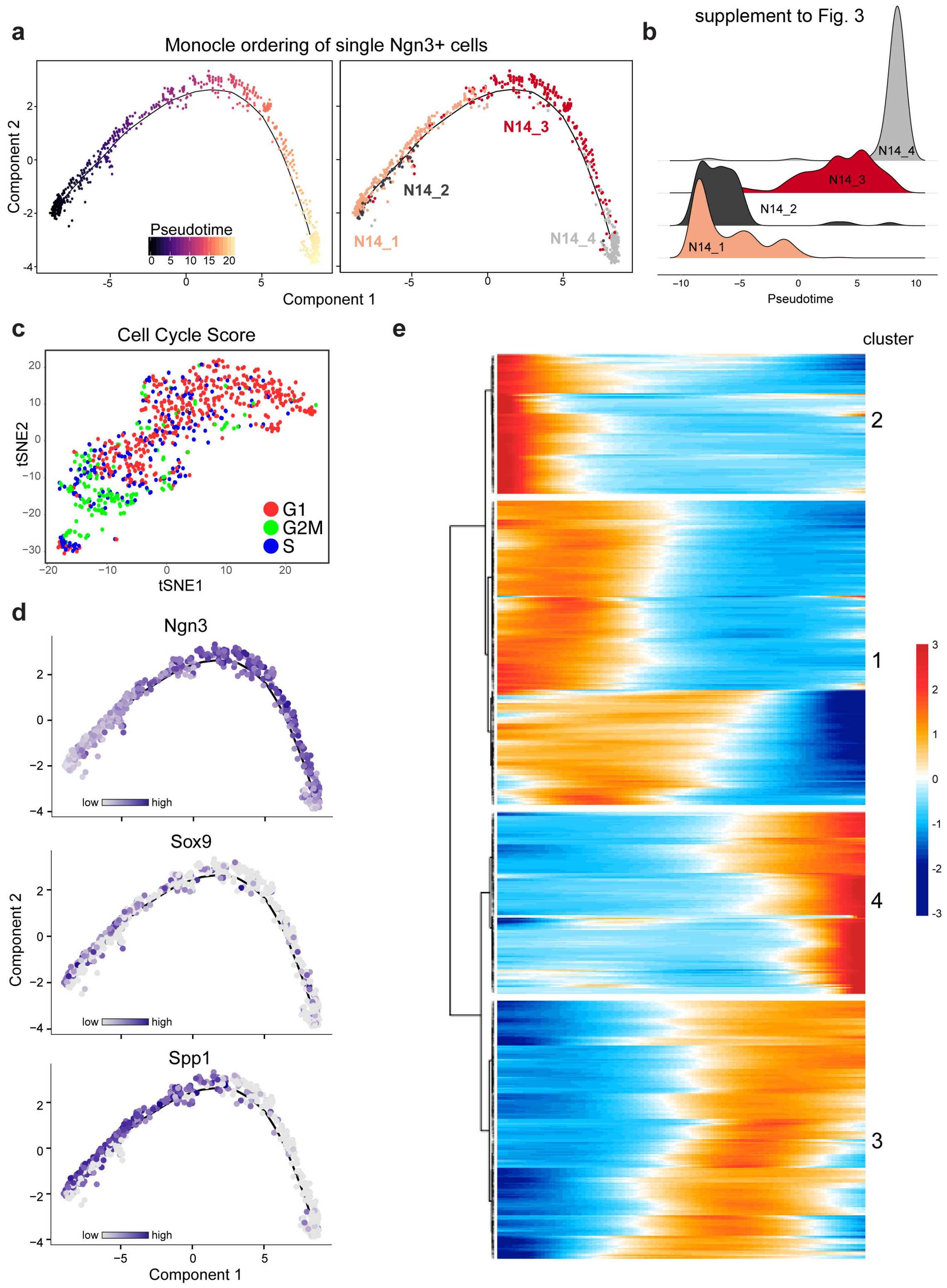

Supplementary Figure 9. Pseudotime and cell cycle analysis of e14.5 *Ngn3*+ cells.

a. Ordering of single e14.5 *Ngn3*+ cells through pseudotime using Monocle. On the left, single cells are shown colored by pseudotemporal ordering. On the right, cells ordered through pseudotime are depicted by *Ngn3*+ cell subtype.

b. Joy plot showing abundance of *Ngn3*+ cell subtypes through pseudotime using Monocle.

c. Cell cycle phase analysis of e14.5 *Ngn3*+ cells. The approximate cell cycle phase was calculated by scoring individual cells on their expression for S-phase genes, G1 genes, and G2M transition genes as defined by Kowalczyk et al (2015)[1].

d. Expression of *Ngn3*, *Sox9*, and *Spp1* projected on the Monocle pseudotime trajectory.

e. Heatmap showing kinetic clusters. Genes with similar gene expression trends over pseudotime group together, derived from Monocle.

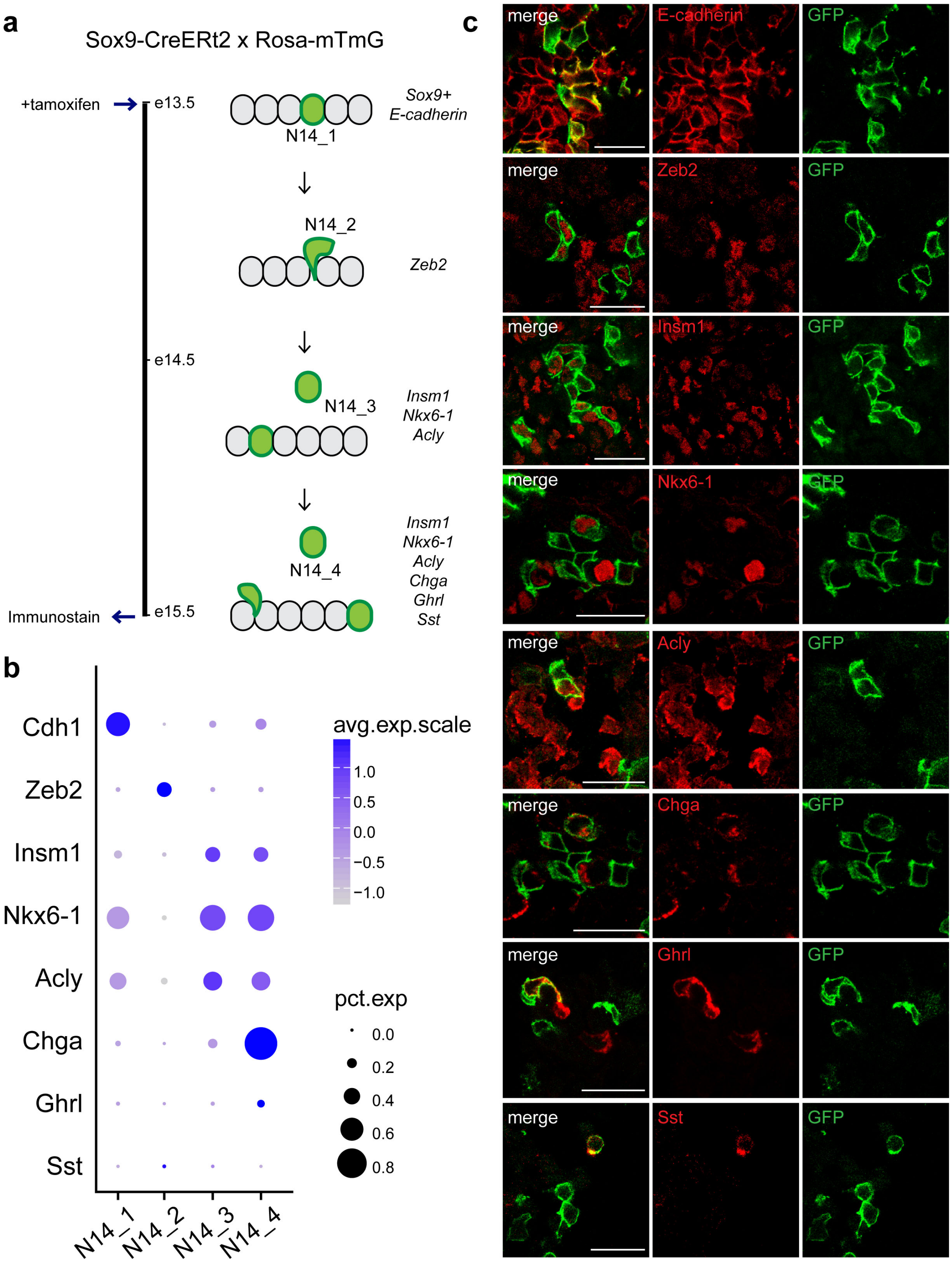

Supplementary Figure 10. Early *Ngn3*+ cell subtype N14_1 matures into N14_2, N14_3, and N14_4 cells.

a. Scheme showing pulse-chase lineage tracing experimental design. Mice were administered tamoxifen at e13.5 or e15.5 to induce Cre recombination of Rosa-mTmG in Sox9+ cells before collecting tissue 48 hours later at e15.5 or e17.5. Candidate markers used for immunostaining are shown to the right for each stage.
b. Dot plot showing gene expression of candidate markers from scRNA-seq.
c. Immunostaining of mouse tissue for Sox9+ cells and their progeny (GFP, in green) at e15.5 (E-cadherin, Zeb2, Insm1, and Sst in red) and e17.5 (Nkx6-1, Acly, Ghrl, and Chga in red). Scale bars=20um.

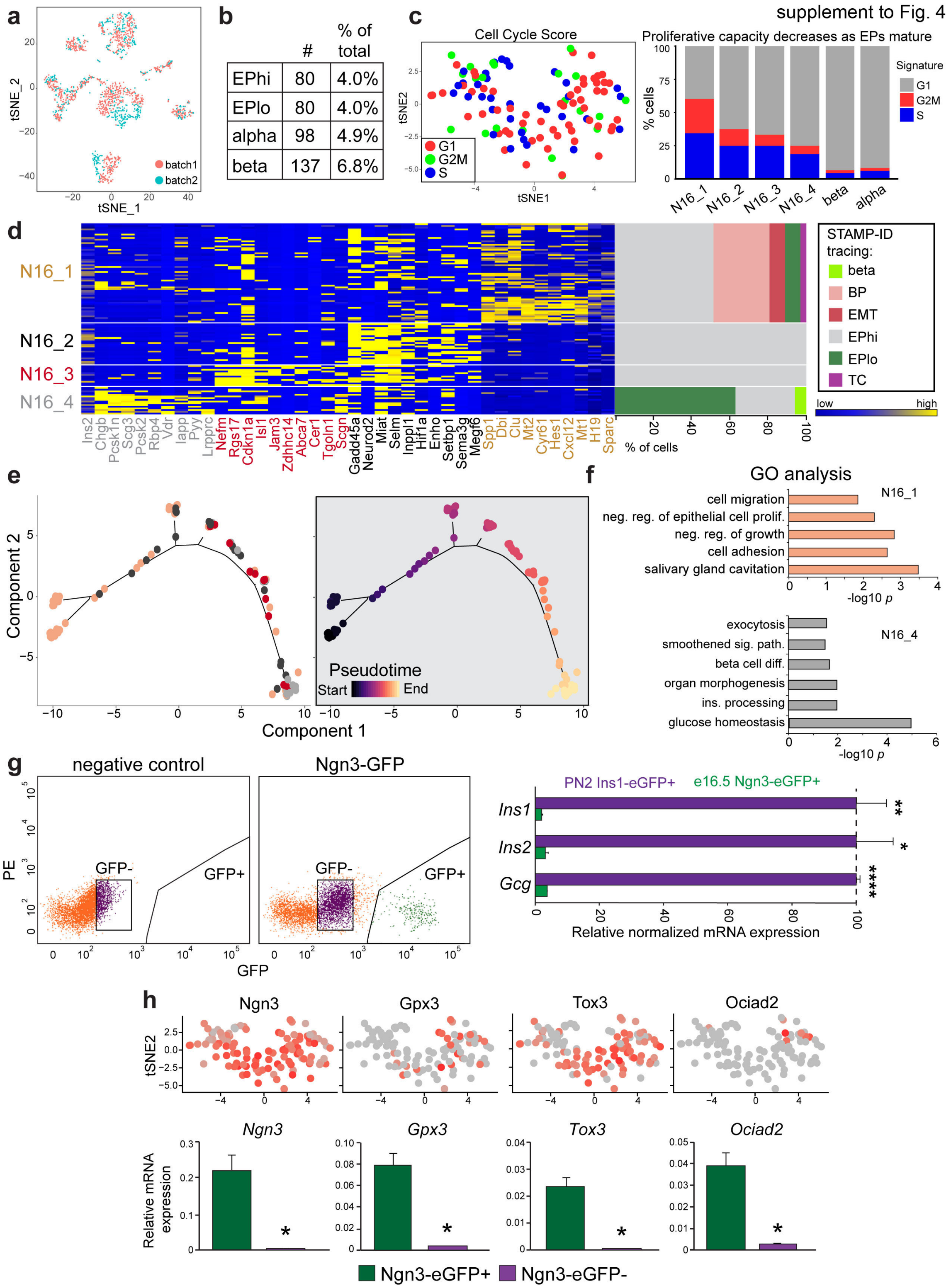

Supplementary Figure 11. ***Ngn3+*** **cell subtypes and pseudotime analysis at e16.5.**

a. Batch analysis of e16.5 droplet-based scRNA-seq. Batch 1 is composed of 12 embryos from a single litter while batch 2 is composed of 9 embryos from a second litter.

b. Number and percentage of EPhi, EPlo, alpha, and beta cells from Fig. 4a.

c. Cell cycle phase analysis of e16.5 *Ngn3*+ cells. The approximate cell cycle phase was calculated by scoring individual cells on their expression for S-phase genes, G1 genes, and G2M transition genes as defined by Kowalczyk et al (2015)[1].

d. Four e16.5 *Ngn3*+ cell subtypes are transcriptionally distinct and represent different developmental stages. Heatmap showing top 10 differentially expressed genes (x-axis) of single *Ngn3*+ cells (y-axis). Expression ranges from blue (low) to high (yellow). STAMP ID tracing of percent composition for each *Ngn3*+ cell subtype is shown to the right.

e. Ordering of single e16.5 *Ngn3*+ cells through pseudotime using Monocle. On the left, cells ordered through pseudotime are depicted by *Ngn3*+ cell subtype. On the right, single cells are shown colored by pseudotemporal ordering.

f. N16 clusters reflect stages of *Ngn3*+ cell maturation. Gene ontology (GO) analysis reveals enriched functions in N16_1 and N16_4.

g. Fluorescence-activated cell sorting (FACS) plots showing e16.5 Ngn3-eGFP mice. Post-sort purity check of isolated Ngn3-eGFP (GFP) cells, showing cells to be 96.7% eGFP+. qPCR shows the expression of *Ins1, Ins2,* and *Gcg* in e16.5 Ngn3-eGFP+ cells relative to Ins1-eGFP. Samples are normalized to *Gapdh.* N=3 biological replicates, N=2 technical replicates. Error bars are SEM. *p<0.05, **p<0.01, ****p<0.001.

h. Droplet-based RNA-seq revealed pancreatic genes not yet described in embryogenesis. Feature plots show candidate gene expression while qPCR shows the expression of *Ngn3, Gpx3, Tox3,* and *Ociad2* in e16.5 EPs and non-EPs. Samples are normalized to *Gapdh.* N=3 biological replicates, N=2 technical replicates. Error bars are SEM. *p<0.05.

supplement to Fig. 4

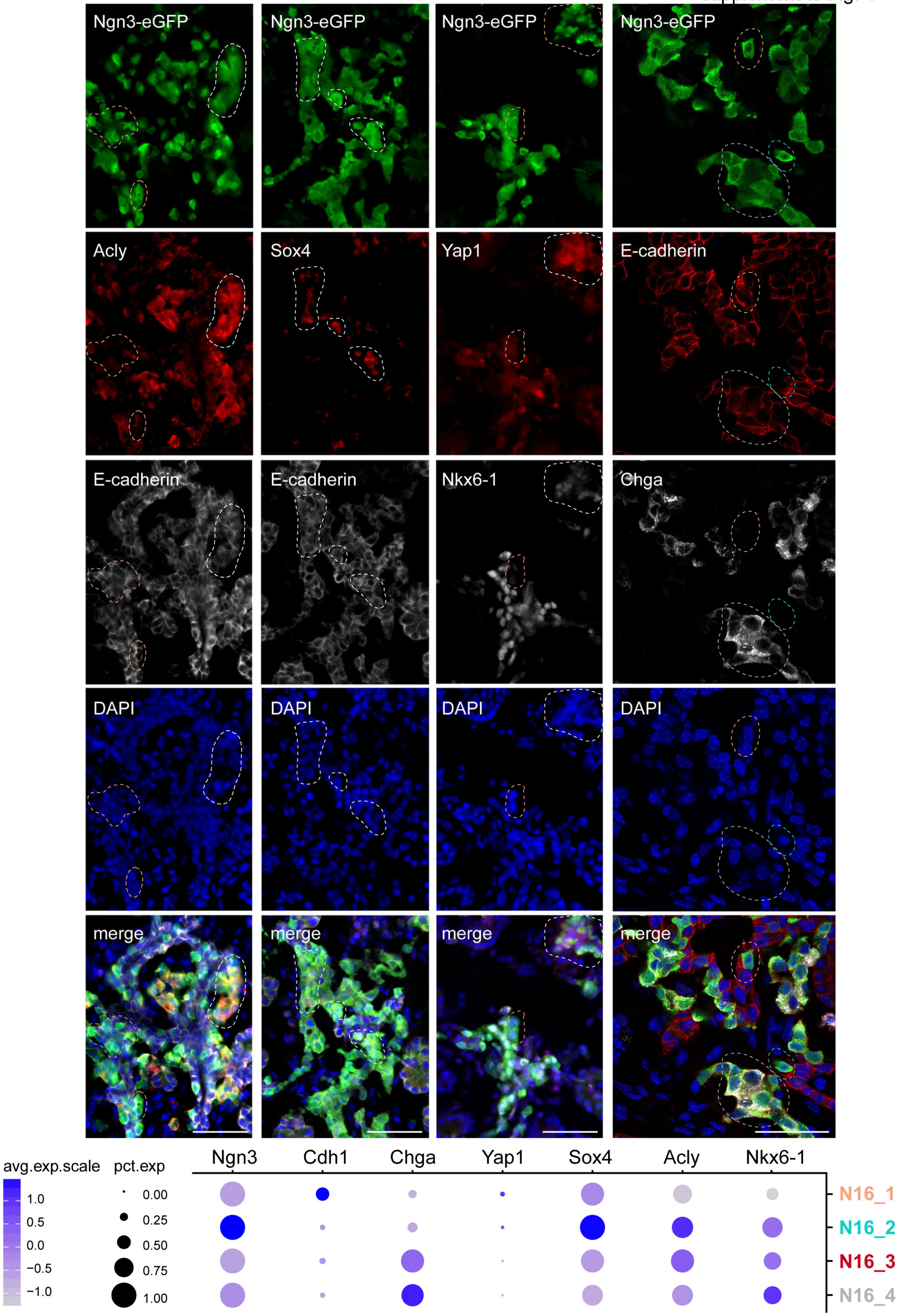

Supplementary Figure 12. Validation of e16.5 *Ngn3*+ cell subtypes.

Immunostaining of e16.5 mouse pancreatic sections (top). *Ngn3+* subtypes are circled by color (N16_1 in tan, N16_2 in teal, N16_3 in maroon, and N16_4 in gray). Scale bar = 50um. Dot plot showing gene expression in *Ngn3+* cell subtypes (bottom).

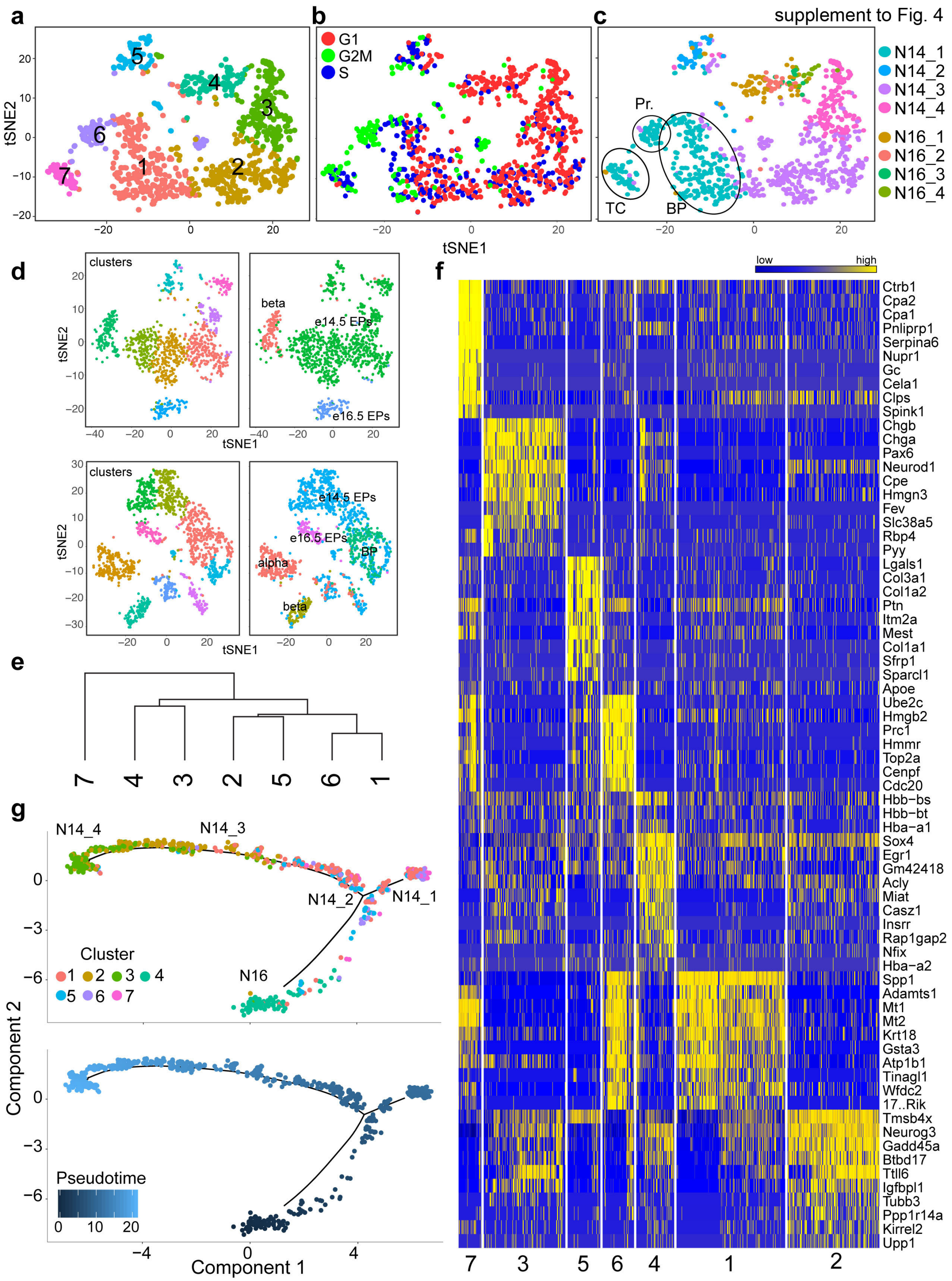

Supplementary Figure 13. Merged single *Ngn3*+ cell transcriptomes from e14.5 and e16.5 show that *Ngn3*+ cells are temporally distinct.

a. tSNE representation of merged single *Ngn3+* cell transcriptomes from e14.5 and e16.5.

b. Cell cycle phase analysis of merged e14.5 and e16.5 EPs. The approximate cell cycle phase was calculated by scoring individual cells on their expression for S-phase genes, G1 genes, and G2M transition genes as defined by Kowalczyk et al (2015)[1].

c. tSNE representation of merged single *Ngn3+* cell transcriptomes from e14.5 and e16.5, tracing the original cluster identity. EP16 cells cluster into cluster 4 from Fig. 6a.

d. *Ngn3+* cells are divergent even when compared to distinct cell types including BPs, alpha cells, and beta cells. On the left, clustering by Seurat. On the right, original cell identity.

e. Dendogram showing the relationships between merged e14.5 and e16.5 Ngn3+ clusters.

f. Merged EP subtypes are transcriptionally distinct. Heatmap showing top 10 differentially expressed genes (x-axis) of single e14.5 and e16.5 *Ngn3+* cells (y-axis). Expression ranges from blue (low) to high (yellow).

g. Ordering of single merged e14.5 and e16.5 *Ngn3+* cells through pseudotime using Monocle. At the top of the panel, cells ordered through pseudotime are depicted by EP subtype. At the bottom, single cells are shown colored by pseudotemporal ordering.

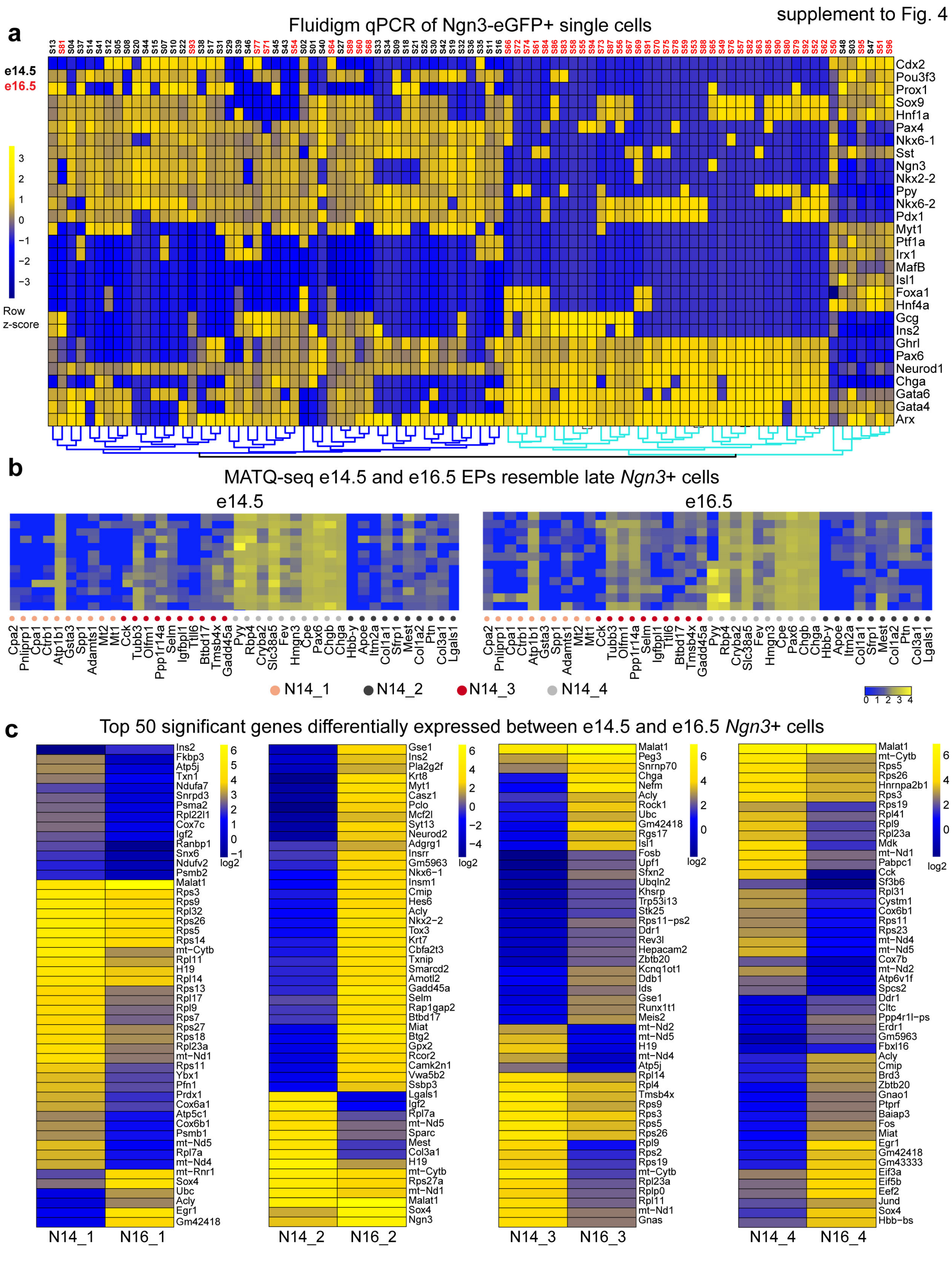

Supplementary Figure 14. Fluidigm C1 analysis, MATQ-seq, and Drop-seq show that EPs from e14.5 and e16.5 are distinct.

a. Single EPs from e14.5 and e16.5 cluster apart. Heatmap from Fluidigm single cell qPCRs from e14.5 and e16.5 Ngn3-GFP+ cells. A total of 92 cells at e14.5 and 138 cells at e16.5 were assessed. Gene expression is shown on the y-axis while single cells are shown on the x-axis.

b. Heatmap showing gene expression from MATQ-seq corresponding to those shown in by droplet-based RNA-seq at e14.5. Dot color below indicate population identity.

c. Comparison of e14.5 and e16.5 *Ngn3+* cell subtypes. Heatmaps show top 50 most differentially expressed (adj. p value) genes by log2 expression.

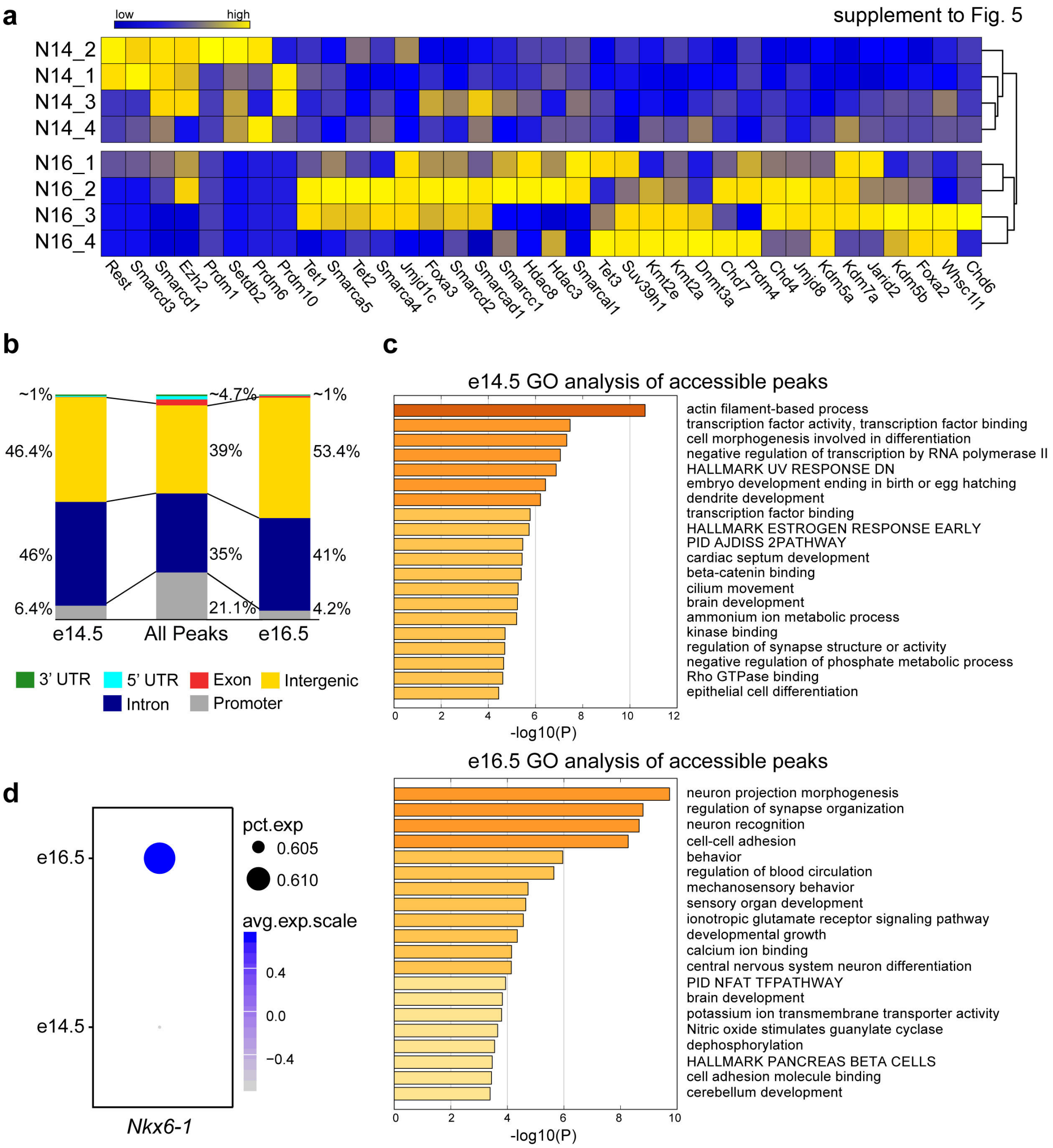

Supplementary Figure 15. Chromatin dynamics change in EPs from e14.5 to e16.5.

a. Heatmap showing gene expression from droplet-based single cell RNA-seq of EP subtypes at e14.5 and e16.5 for select epigenetic related genes.

b. Annotation of ATAC peaks from e14.5 and e16.5 Ngn3-eGFP+ EPs.

c. Gene ontology (GO) analysis of enriched accessible peaks in e14.5 and e16.5 Ngn3-eGFP+ cells.

d. Expression of *Nkx6-1* by droplet-based scRNA-seq in *Ngn3*+ e14.5 and e16.5 cells.

supplement to Fig. 7

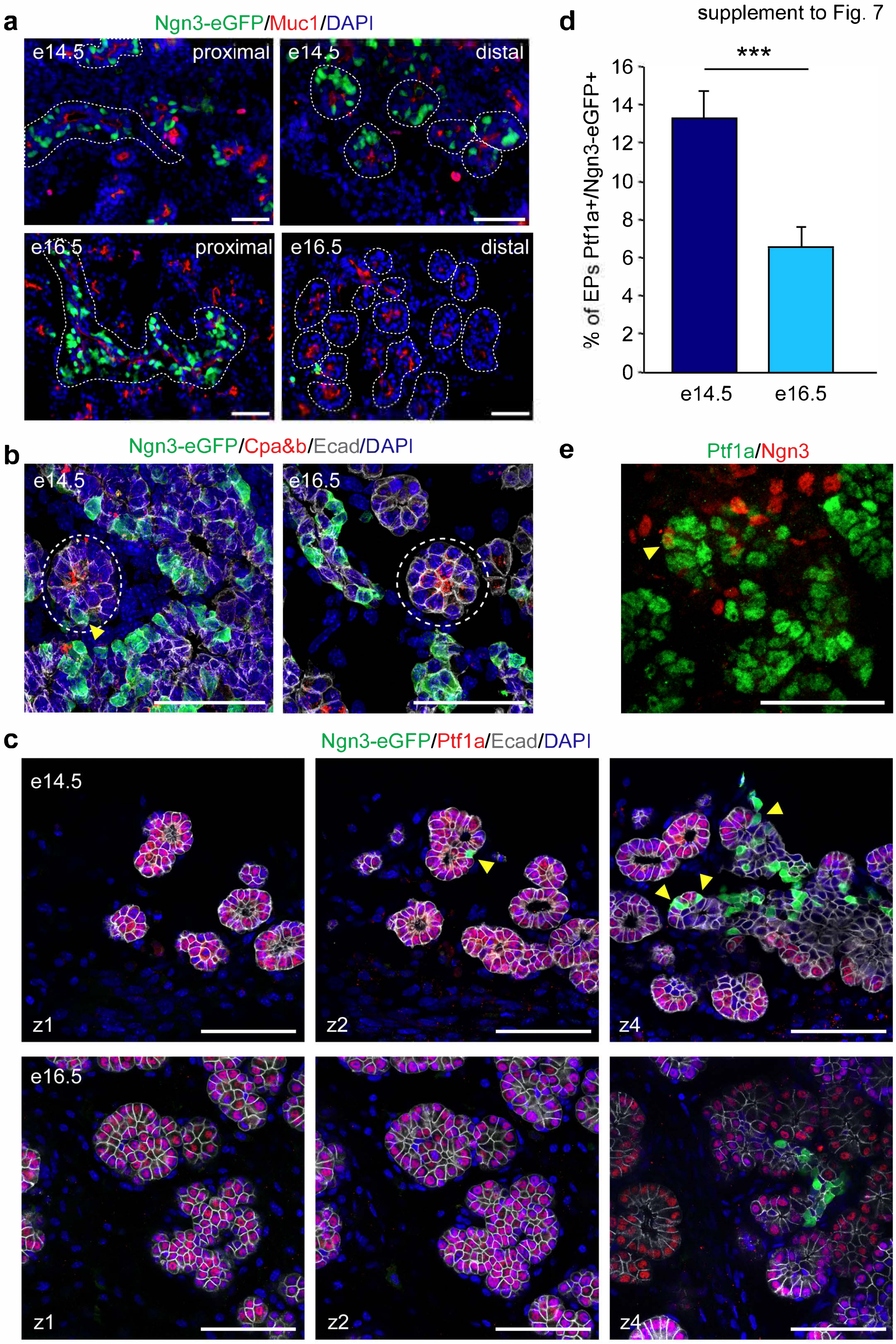

Supplementary Figure 16. EPs form in e14.5 TCs but not in e16.5 TCs.

a. Immunostaining of e14.5 and e16.5 pancreas for EPs (Ngn3-eGFP in green), apical epithelium (Muc1 in red), and nuclei (DAPI in blue). Proximal and distal regions of tissue are shown with white dashed lines outlining trunk and tip regions. Scale bars=50um.

b. Immunostaining of e14.5 and e16.5 pancreas for EPs (Ngn3-eGFP in green), TCs (Cpa&b in red), epithelium (Ecad in white), and nuclei (DAPI in blue). White dashed line outlines a cluster of tip cells. Scale bars=50um.

c. Z-sections from whole mount immunostained optically cleared tissue at e14.5 and e16.5. EPs are shown in green (Ngn3-eGFP), TCs in red (Ptf1a), epithelium in gray (Ecad), and nuclei in blue (DAPI). Scale bars=50um.

d. Quantification of EPs in TCs. Ngn3-eGFP and Ptf1a co-stained cells were counted before counting total Ngn3-eGFP+ cells to determine the percentage of EPs that were Ptf1a+. N=6 biological replicates and 22 sections at e14.5; N=5 biological replicates and 12 sections at e16.5. ***p<0.005.

e. Immunostaining of e14.5 pancreas for Ngn3 protein (in red) and Ptf1a to mark TCs (in green). Yellow arrow indicates cell co-expressing both markers. Scale bar=50um.

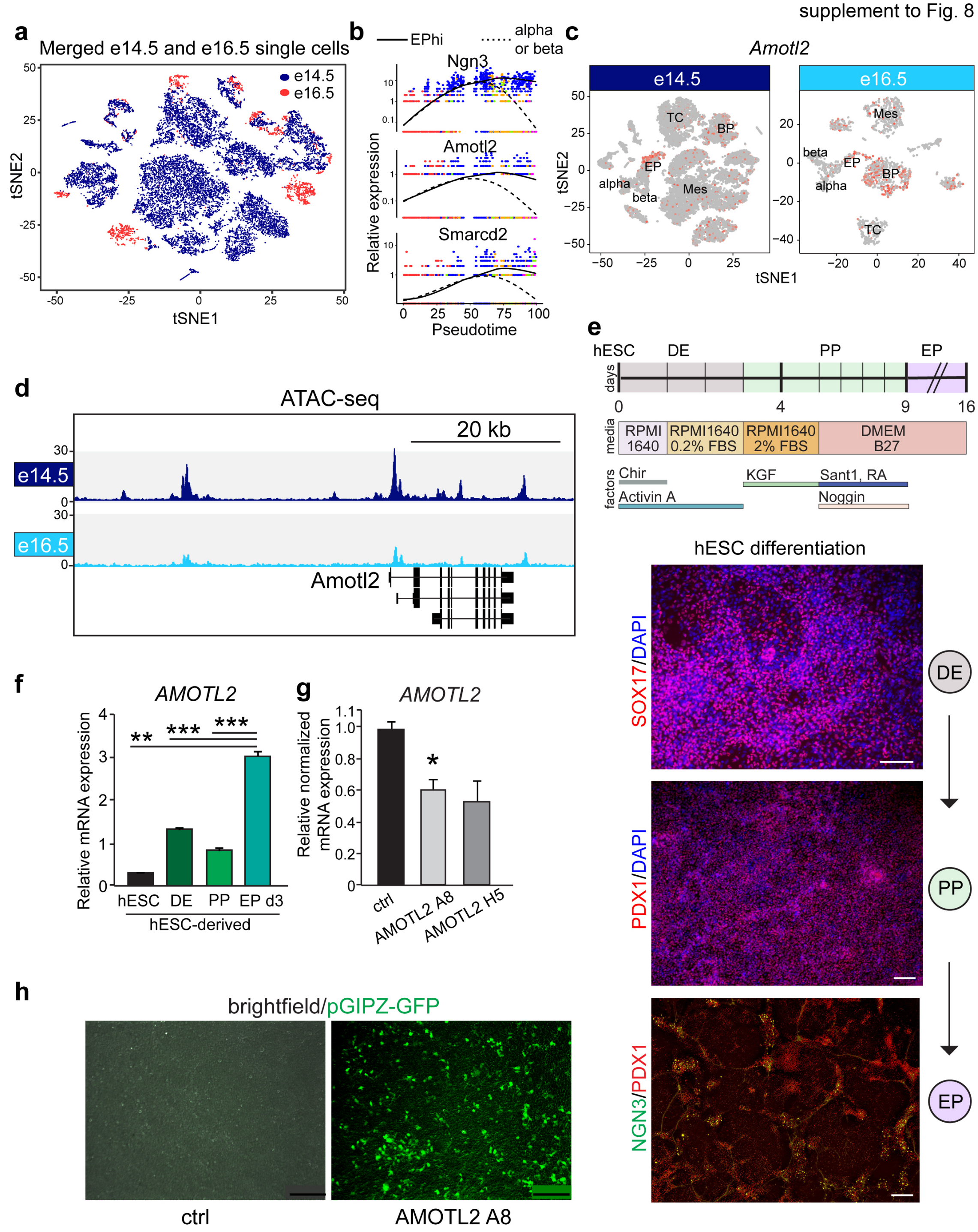

Supplementary Figure 17. AMOTL2 loss-of-function in hPSC-derived pancreatic cells.

a. tSNE representation of merged e14.5 and e16.5 single cells from the whole pancreas. Blue dots are cells are from e14.5 while red dots are cells from e16.5.

b. Pseudotime plots are shown on the right of Ngn3, Amotl2, and Smarcd2, with the solid line showing EPhi cells (early EPs) and the dotted line showing alpha or beta cells.

c. tSNE representation of single cells expressing *Amotl2* at e14.5 and e16.5 in EPs, with gray indicating no expression and red indicating high expression.

d. ATAC-seq profiles of *Amotl2* from e14.5 (shown in navy) and e16.5 (shown in light blue) EPs, after normalization and combination of all biological replicates. Genomic loci: chr9:102,577,350-102,643,781

e. Differentiation of hESCs into pancreatic cells. On the top, differentiation scheme. Below, immunostaining of SOX17, PDX1, and NGN3 shows a representation of hESC differentiation to the definitive endoderm (DE), pancreatic progenitor (PP), and EP stages. ~95% of cells express SOX17 at definitive endoderm stage, ~90% express PDX1 at the PP stage, while ~50% of cells express NGN3 at the EP stage. Scale bars=100um

f. qPCR showing *AMOTL2* expression in human *in vitro* pancreatic directed differentiation. d3, day 3. N=3 biological replicates, N=2 technical replicates. Scale bars are SEM, **$p<0.01$, ***$p<0.005$ compared to EP d3.

g. *AMOTL2* expression using two different shRNAs, with A8 providing the most efficient knockdown. N=3 biological replicates, N=2 technical replicates. Error bars are SEM. *$p<0.05$.

h. Viral efficiency of pGIPZ AMOTL2 A8 shRNA as indicated by GFP expression.

supplement to Fig. 9

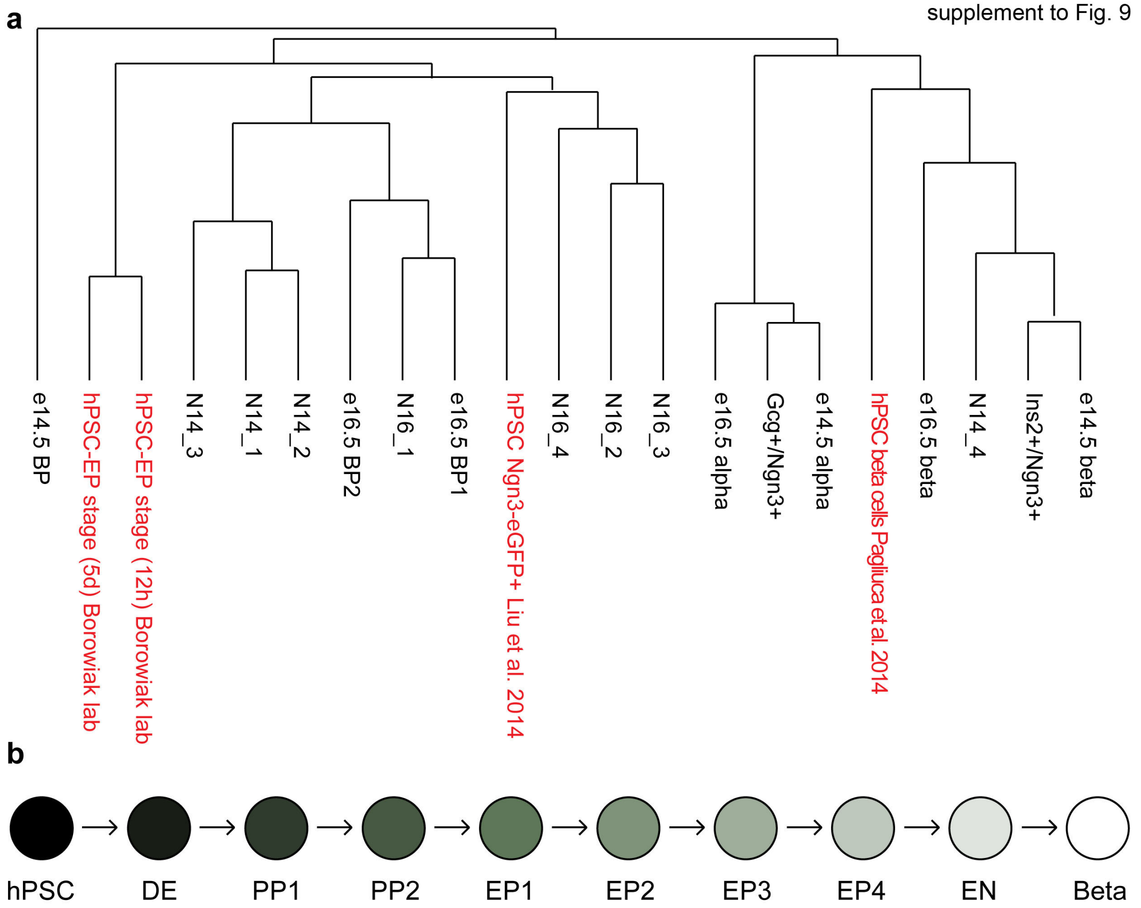

Supplementary Figure 18. Comparing the fidelity of *in vitro* hPSC-derived pancreatic cells to *in vivo* murine cells.

a. Dendogram showing the relationship of hPSC-derived pancreatic cells from various labs to *in vivo* murine pancreatic cell types. hPSC-EP stage cells 12 hours (12h) or 5 (5d) days after the start of the EP stage are related to e16.5 *Ngn3*+ cells. hPSC-derived EP stage Ngn3-GFP+ cells from Liu et al. (2014)[2] very closely resemble late e16.5 *Ngn3*+ cells, indicating these cells have a high potential for the beta cell fate. hPSC-derived beta cell stage cells from Pagliuca et al. (2014)[3] resemble murine beta cells.

b. New differentiation scheme to direct hPSCs to the beta cell fate, with four distinct phases of EP stage cells.

Supplementary Table 1. Primary antibody information.

Antibody	Cat. #	Source	Dilution
Ins	A056401	Dako	1:1000
Gcg	sc-7779	SCBT	1:200
Cpa1/Cpa2/Cpb	ab181146	Abcam	1:100
Ngn3	N/A	Maike Sander	1:2000
Vim	AB5733	Millipore	1:400
Dcn	6D6	DSHB	1:100
Ptf1a	N/A	Christopher Wright	1:10,000
Ociad2	NBP1-77085	NovusBio	1:20
Ppp1r1a	ab40877	Abcam	1:100
Dcdc2	ab157186	Abcam	1:100
Rbfox2	N/A	Thomas Cooper	1:500
GFP	ab13970	Abcam	1:1000
Chga	ab15160	Abcam	1:100
Zeb2	sc-271984	SCBT	1:100
Neurod1	sc-1084	SCBT	1:100
Nkx6-1	F55A10	DSHB	1:100
E-cadherin	AF-748	R&D Systems	1:500
Mucin1	HM1630P0	Fisher Scientific	1:400
Acly	NBP2-37429	Novus Bio	1:50
Sox4	N/A	Michael Wegner	1:500
Stat3	4904S	CST	1:100
Nfia	HPA006-111	Sigma Aldrich	1:100
Col4a1	AB756P	Millipore	1:100

Yap1	NB110-58358	Novus Bio	1:100
Rab7	ab50533	Abcam	1:100
NGN3	RES4129	BCBC	1:100
SOX17	BAF1924	R&D Systems	1:100
PDX1	R&D Systems	AF2419	1:100

Supplementary Table 2. Primer sequences.

Gene	Species	f (5'-3')	r (5'-3')
Gapdh	Mouse	aatgtgtccgtcgtggatctga	agtgtagcccaagatgcccttc
Ngn3	Mouse	aggttgttgtgtctctgggg	gtcactgactgacctgctgc
Ins1	Mouse	ggagcgtggcttcttctaca	ctgcagcactgatccacaat
Ins2	Mouse	tctacaatgccaccacgcttctg	tttgtcaagcagcacctttg
Gcg	Mouse	tggtgctcatctcgtcagag	aacaacattgccaaacgtca
Sst	Mouse	ttctctgtctggttgggctc	cagactccgtcagtttctgc
MafB	Mouse	cacacgtagcaagaggtgga	agacaggctttgcgtcctaa
MafA	Mouse	ctctggagctggcacttctc	aggaggtcatccgactgaaa
Gpx3	Mouse	atggtaccactcataccgcc	catcctgccttctgtccct
Tox3	Mouse	gtggaatgtctgctcactgg	agtgcctggggtactacgg
Btg2	Mouse	tagccagaacctttggatgg	gcgagcagagactcaaggtt
Ociad2	Mouse	tctcctctgtggatgtgcag	agtgtccactcatgggaacc
Malat1	Mouse	caggaaggtgaagaaaaagctg	gaatgctggcatccaaagtt
TBP	Human	tgtgcacaggagccaagagt	attttcttgctgccagtctgg
INS	Human	agcctttgtgaaccaacacc	gctggtagagggagcagatg
AMOTL2	Human	catctctcgctccagcttct	cctggcaagcaagacacag
GCG	Human	aagcatttactttgtggctggatt	tgatctggatttctcctctgtgtct

Supplementary references

1. Kowalczyk, M.S. et al. Single-cell RNA-seq reveals changes in cell cycle and differentiation programs upon aging of hematopoietic stem cells. Genome Res 25, 1860-1872, doi:10.1101/gr.192237.115 (2015)

2. Liu, H. et al. Systematically labeling developmental stage-specific genes for the study of pancreatic beta-cell differentiation from human embryonic stem cells. Cell Res 24, 1181-1200, doi:10.1038/cr.2014.118 (2014).

3. Pagliuca, F.W. et al. Generation of functional human pancreatic beta cells in vitro. Cell 159, 428-439, doi:10.1016/j.cell.2014.09.040 (2014).
