# Supplementary material for: Endocrine lineage biases arise in temporally distinct endocrine progenitors during pancreatic morphogenesis
Source: Nat Commun. 2018 Aug 22;9:3356. doi: 10.1038/s41467-018-05740-1 (PMC6105717; doi:10.1038/s41467-018-05740-1)
Supplement: Supplementary file 3 — Description of Additional Supplementary Files [file 41467_2018_5740_MOESM3_ESM.docx]

**Description of Additional Supplementary Files**

File Name: Supplementary Data 1

Description: Enriched genes in different subtypes of e14.5 pancreatic cells.

File Name: Supplementary Data 2

Description: Enriched genes in e14.5 Ngn3+ cell subtypes.

File Name: Supplementary Data 3

Description: Kinetic clusters of genes changing in e14.5 Ngn3+ cells over pseudotime. 2

File Name: Supplementary Data 4

Description: Comparison of genes changing in e14.5 mouse Ngn3+ cell maturation compared to human EP development.

File Name: Supplementary Data 5

Description: Enriched genes in different subtypes of e16.5 pancreatic cells.

File Name: Supplementary Data 6

Description: Enriched genes in e16.5 Ngn3+ cell subtypes.

File Name: Supplementary Data 7

Description: Kinetic clusters of genes changing in merged e14.5 and e16.5 Ngn3+ cells over pseudotime.

File Name: Supplementary Data 8

Description: Enriched e14.5 ATAC-seq peaks.

File Name: Supplementary Data 9

Description: Annotation of enriched e14.5 ATAC-seq peaks.

File Name: Supplementary Data 10

Description: Enriched e16.5 ATAC-seq peaks.

File Name: Supplementary Data 11

Description: Annotation of enriched e16.5 ATAC-seq peaks.
